# Role of Antioxidant Enzymes and Glutathione S-Transferase in Bromoxynil Herbicide Stress Tolerance in Wheat Plants

**DOI:** 10.3390/plants11202679

**Published:** 2022-10-12

**Authors:** Reda M. Gaafar, Mohamed El-Anwar H. Osman, Atef M. Abo-Shady, Ibrahim A. A. Almohisen, Ghada Ahmed Badawy, Maysa M. F. El-Nagar, Gehan A. Ismail

**Affiliations:** 1 Botany Department, Faculty of Science, Tanta University, Tanta 31527, Egypt; 2 Department of Biology, Faculty of Science and Humanities, Shaqra University, Quwayiyah 11971, Saudi Arabia; 3 Department of Biology, University College of Umluj, Umluj Branch Tabuk University, Tabuk 71491, Saudi Arabia; 4 Department of Botany, Faculty of Science, El-Fayoum University, Fayoum 63514, Egypt

**Keywords:** *Arthrospira platensis*, *Nostoc muscorum*, herbicide safeners, *Triticum aestivum*, vegetation stage, metabolism, qRT-PCR

## Abstract

Background: Numerous pesticides and herbicides used in excess cause oxidative stress in plants. These chemicals protect plants from weeds and pests, but they also have very negative side effects, making them common abiotic stressors. One of the most significant nutritional crops in the world is the wheat plant. Conditions of herbicide stress have a negative impact on the plant’s phonological phases and metabolic pathways. Plants primarily make an effort to adjust to the environment and develop oxidative homeostasis, which supports stress tolerance. Methods: When controlling broadleaf weeds that emerge after cereal crop plants have been planted, bromoxynil is frequently used as a selective-contact herbicide. This study looked at the effects of the cyanobacteria *Arthrospira platensis* and *Nostoc muscorum* aqueous extracts, tryptophan, and bromoxynil (Bh) alone or in combination on wheat plant growth parameters. Both tryptophan and cyanobacterial extract were used as chemical and natural safeners against Bh application. The antioxidant activity and transcriptome studies using qRT-PCR were assayed after 24, 48, 72, 96 h, and 15 days from Bh application in the vegetation stage of wheat plants (55 days old). Results: In comparison with plants treated with Bh, wheat plants treated with cyanobacteria and tryptophan showed improvements in all growth parameters. Following application of Bh, wheat plants showed reduced glutathione content, as well as reduced antioxidant enzyme activities of superoxide dismutase, catalase, glutathione peroxidase, and glutathione-s-transferase. The combination of different treatments and Bh caused alleviation of the harmful effect induced by Bh on the measured parameters. Additionally, the expression of glutathione synthase and glutathione peroxidase, in addition to those of three genes (*Zeta*, *Tau*, and *Lambda*) of the GST gene family, was significantly upregulated when using Bh alone or in combination with different treatments, particularly after 24 h of treatment. Conclusion: The current study suggests using cyanobacterial extracts, particularly the *A. platensis* extract, for the development of an antioxidant defense system against herbicide toxicity, which would improve the metabolic response of developed wheat plants.

## 1. Introduction

Weeds are an important factor influencing crop production through competition for environmental resources (light, water, and nutrients in the soil) and cause heavy yield losses [1]. According to Nishimoto [2], weeds cause 14% of the damage to global agricultural production, and this may lead to crop yield losses of 25–30% in developing nations. Consequently, demand for the use of herbicides has grown and has become one of the most prominent ways to combat weeds [3]. Herbicides are biologically active xenobiotic compounds and have been established as a primary method for weed species control in modern agriculture practices [4]. Herbicide use is known to increase crop yield, but when used excessively, especially with sensitive crops, it may disrupt normal biochemical and physiological processes and put crops at risk [5]. As a secondary effect of herbicide application, many studies have shown that reactive oxygen species (ROS) can accumulate in plant cells [4,5]. Excess ROS can damage plant cells by causing membrane lipid and protein oxidation, enzyme activity inhibition, DNA and RNA damage, etc., and can even result in cell death [6]. Due to herbicide toxicity, chemical compounds called safeners were developed to increase crop selectivity [7]. Herbicide safeners are chemical agents that increase the tolerance of monocotyledonous cereal plants to herbicides without affecting the efficacy of weed control [8]. Chemical safeners have been used on many cereal crops, such as wheat [9], sorghum [10], rice [11], and maize [12]. Safeners act to decrease plant–herbicide sensitivity by reducing herbicide translocation and uptake or by accelerating the metabolism of herbicides into less active or inactive compounds [8]. Additionally, safeners could cause a coordinated increase in the intracellular proteins or enzymes involved in the successive phases of multistep xenobiotic metabolic degradation (detoxification system) by triggering an up-regulation of their corresponding gene(s) transcription [13]. Plant glutathione S-transferases (GSTs) are global and multifunctional enzymes that catalyze the detoxification of xenobiotic compounds, especially herbicides, by their conjugation to reduced glutathione (GSH) molecules [14]. 

Unfortunately, the repetition of using chemical safeners in agriculture may cause their accumulation in plants and soils, which can be dangerous to humans and the environment [15]. In the search for more tolerable and environmentlly friendly solutions, biologically based products, such as microalgae and cyanobacteria, have emerged as a valuable source for crop protection from different stresses as well as for improvements in agricultural production [16]. According to the literature, algae and cyanobacterial metabolites (e.g., phenols, terpenoids, amino acids, fatty acids, polysaccharides, and carotenoids) could play an important role in plant protection against biotic and abiotic stress factors [17,18]. In addition, microalgae and cyanobacteria also incorporate phytohormones, which are known for their activity as plant-growth promoters [15,16,17,18,19]. Both biomass and extracts from microalgae and cyanobacteria are currently commercially available for the enhancement of agricultural production [17,18,19,20].

In this study, wheat (*Triticum aestivum* L. var. Giza 171) was selected as a model plant to investigate the induced oxidative stress due to bromoxynil (Bh) application. Bromoxynil is one of the most commonly used herbicides. It is a selective photosynthesis inhibitor (nitrile group) frequently used as a post-emergence herbicide for controlling annual broadleaf weeds that grow with cereal crops, including wheat, barley, oats, and sweet corn. Therefore, the current study aimed to investigate the activities of antioxidant enzymes in wheat plants in response to Bh treatment alone or in combination with synthetic tryptophan or natural cyanobacterial extract treatments as herbicide safeners. We also explored the genes that were related to the mode of action by which wheat plants develop protection against Bh toxicity.

## 2. Results

### 2.1. Growth Parameters

Results presented in Table 1 reveal the changes in growth parameters that occurred in the wheat plant in response to the different treatments. The results indicated that the Bh treatment induced a significant reduction in plant height (23.56%), plant fresh weight (34.19%), and plant dry weight (42.39%) parameters compared with the values recorded for the control plants. Alternatively, the combination of Bh with different treatments improved the values of the studied growth parameters. The best results were in response to the *A. platensis* *+* Bh treatment, recording 9.04% for plant height, 13.94% for plant fresh weight, and 32.04% for plant dry weight, respectively (Table 1).

### 2.2. Biochemical Analysis

#### Antioxidant Activity 

**Figure 1:**
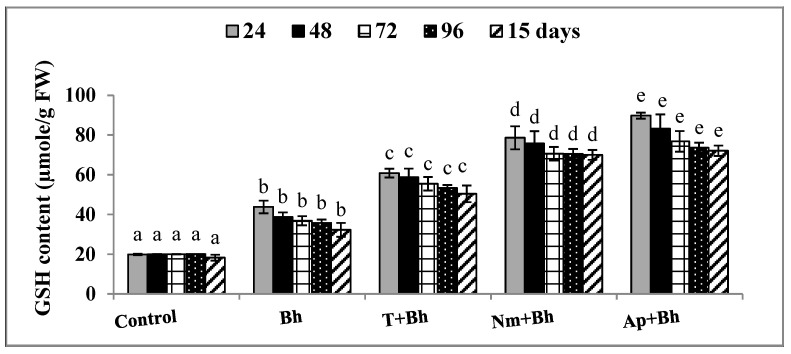
Effects of Bh application alone or in combination with different safener treatments on the GSH content of wheat plants. Data are presented as the mean ± SD (*n* = 5). Bh: bromoxynil; T + Bh: tryptophan + bromoxynil; *Nm +* Bh: *Nostoc muscorum* + bromoxynil; *Ap +* Bh: *Arthrospia platensis* + bromoxynil. Different letters on the bars indicated a significant difference between treatments at *p* < 0.05.

**Figure 2:**
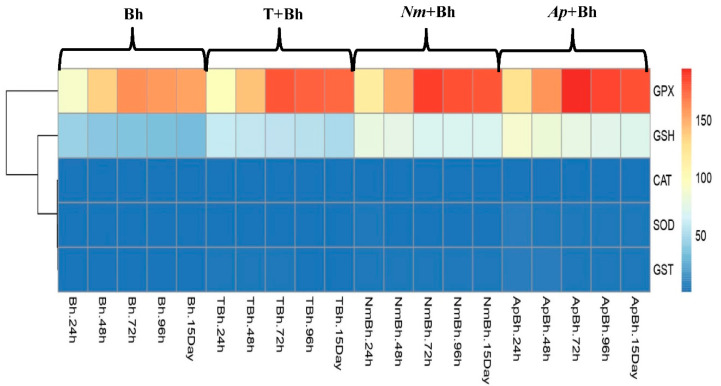
Heat map showing expression patterns of the differential expressed antioxidant-related enzymes as a response to Bh application alone or in combination with different safener treatments on wheat plants at the vegetation stage. The transcript abundance level is shown in the color legend, where red specifies the transcripts with a higher level, and blue specifies transcripts with a lower level. Bh: bromoxynil; T + Bh: tryptophan + bromoxynil; *Nm +* Bh: *Nostoc muscorum* + bromoxynil; *Ap +* Bh: *Arthrospia platensis* + bromoxynil.

The activities of SOD, CAT, GPX, GST, and GSH content in the wheat leaves varied after 24, 48, 72, 96 h, and 15 days from Bh application. In the presence of Bh alone or in combination with different safeners, SOD, CAT, GPX, and GST activities, and GSH content were estimated (Table 2, Figure 1 and Figure 2). Generally, at each tested time course, there was a significant difference between all treatments; however, a gradual increase in all activities was noticed to reach its maximum values at 24 and 72 h, and then, eventually, decreased at 96 h after Bh application. SOD, GST enzymes, and GSH content recorded their maximum activities at 24 h of application. On the other hand, Bh, CAT, and GPX recorded their maximum activities after 72 h of application for all treatments. Collectively, the maximum activity of all measured antioxidant activities was detected in *Ap +* Bh, compared with Bh application alone or control plants. After 24 h, SOD activity was increased by 17.5%, 51.05%, 1.6 fold, and 2.94 fold for Bh, T + Bh, *Nm +* Bh, and *Ap +* Bh, respectively. Similarly, GST activity was improved by 98.5%, 1.57 fold, 2.53 fold, and 3.36 fold for the same treatments compared with control plants. A higher increase was estimated for GSH content by 1.2 fold, 2.06 fold, 2.95 fold, and 3.5 fold for Bh, T + Bh, *Nm +* Bh, and *Ap +* Bh, respectively (Table 2, Figure 1). 

After 72 h, the same increment pattern was evaluated for CAT enzyme activity, recording 39.3, 65.2, 70.8, and 86.51%, and also for GPX activity, but with a higher magnitude, recording 91.42%, 1.15 fold, 1.24 fold, and 1.32 fold for Bh, T + Bh, *Nm +* Bh, and *Ap +* Bh, respectively, in comparison with control plants (Table 2, Figure 2). 

After 15 days from Bh application, SOD, CAT, GPX, GST enzyme activity, and GSH content were also estimated. In general, all antioxidant activities were intrinsically higher in response to the Bh application, recording 5.04, 32.56, 88.85, 39.53, and 76.68% for SOD, CAT, GPX, and GST enzymes and GSH content, respectively, as compared with control plants (Table 2 and Figure 1). Similarly, SOD, CAT, GPX, GST enzyme activity, and GSH content gradually and significantly increased in wheat plants when treated with T + Bh, then *Nm* + Bh, and finally *Ap* + Bh. In comparison with the values of Bh treatment alone, *Ap* + Bh treatment recorded the highest increases for SOD (1.14 fold), CAT (10.53%), GPX (19%), GST (1.38 fold), and GSH (1.23 fold), respectively. After 15 days from Bh application, the lowest values were recorded in response to T + Bh treatment for SOD (17.8%), CAT (4.39%), GPX (13.4%), GST (38.89%), and GSH (56.27%), respectively (Table 2, Figure 1 and Figure 2).

### 2.3. Genes Expression Analysis

#### Quantitative Real-Time PCR (qRT-PCR) of Antioxidant Enzyme-Related Genes

**Figure 3:**
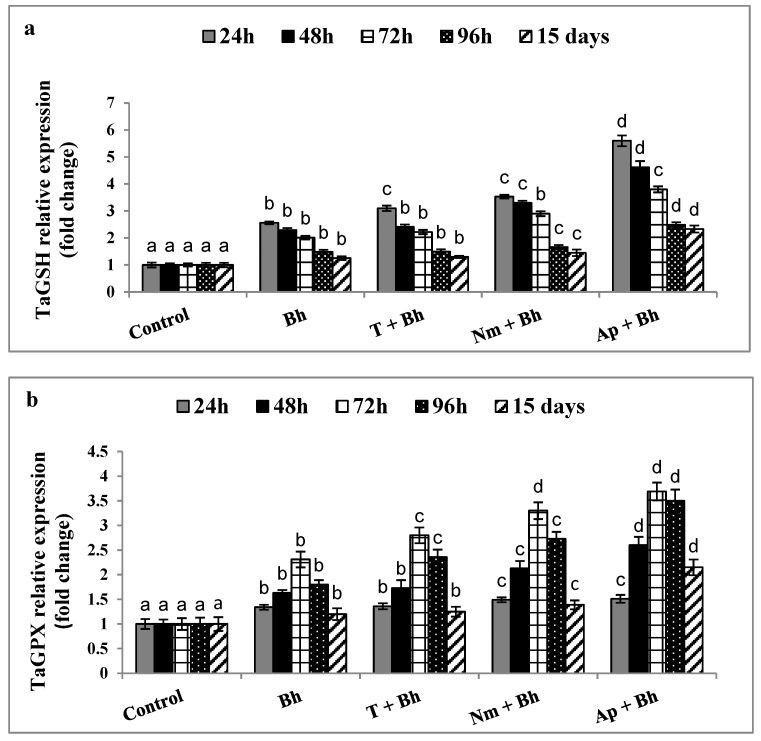
Relative expression of wheat antioxidant-related genes, (**a**) *TaGSH* and (**b**) *TaGPX*, after treatment with bromoxynil alone and or in combination with different safener treatments. The GABDH gene was used as the housekeeping gene. Data are presented as the mean ± SD (*n* = 3). Bh: bromoxynil; T + Bh: tryptophan + bromoxynil; *Nm +* Bh: *Nostoc muscorum* + bromoxynil; *Ap +* Bh: *Arthrospia platensis* + bromoxynil. Different letters on the bars indicated a significant difference between treatments at *p* < 0.05.

**Figure 4:**
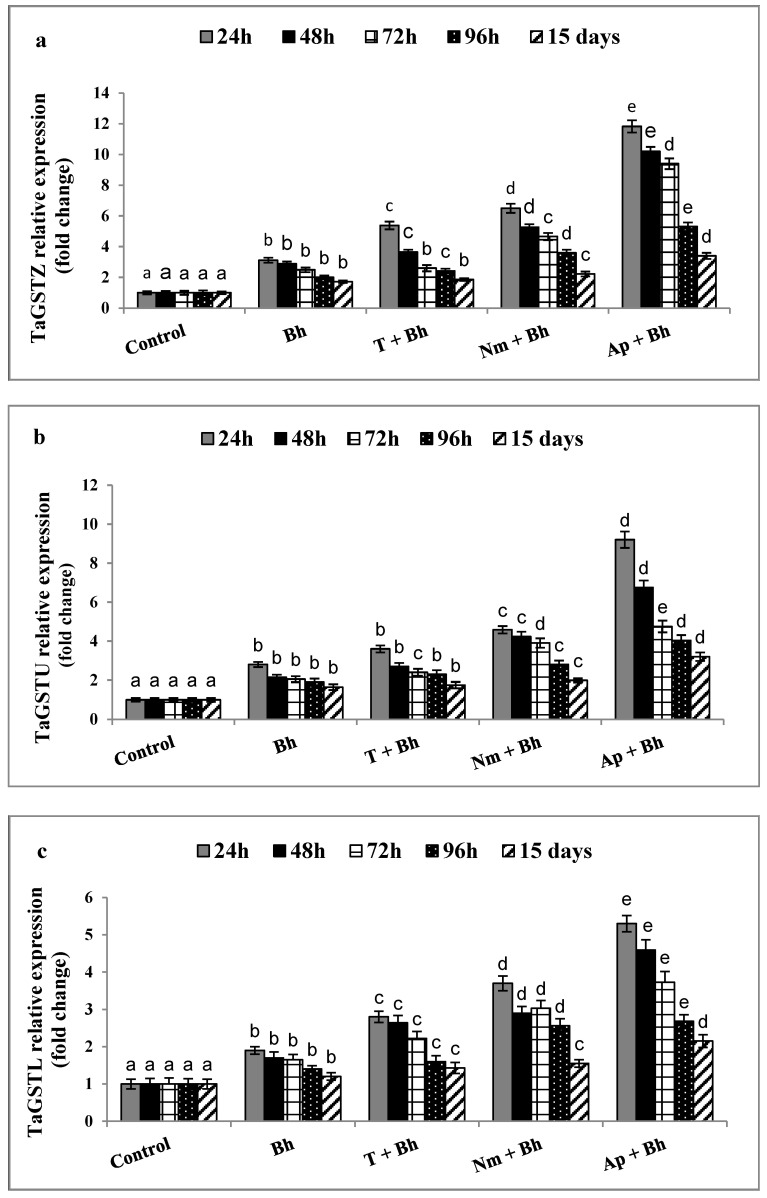
Relative expression of wheat antioxidant-related genes: (**a**) *TaGSTZ*, (**b**) *TaGPTU*, and (**c**) *TaGSTL* after treatment with bromoxynil alone or in combination with different safener treatments. The GABDH gene was used as the housekeeping gene. Data are presented as the mean ± SD (*n* = 3). Bh: bromoxynil; T + Bh: tryptophan + bromoxynil; *Nm +* Bh: *Nostoc muscorum* + bromoxynil; *Ap +* Bh: *Arthrospia platensis* + bromoxynil. Different letters on the bars indicated a significant difference between treatments at *p* < 0.05.

**Figure 5:**
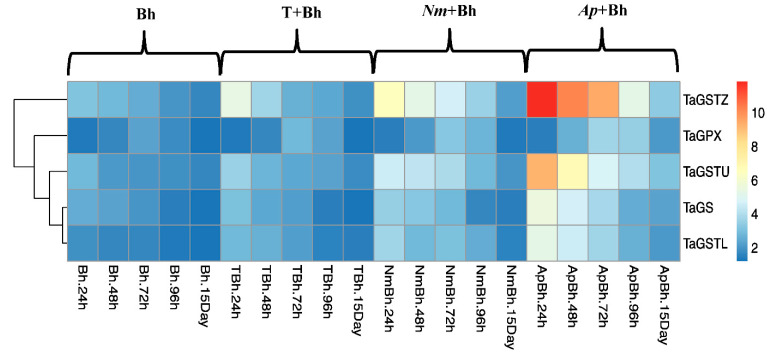
A heatmap showing the effect of Bh application alone or in combination with different safener treatments on wheat plants at the vegetation stage on the relative expression of wheat detoxification-related genes. The transcript abundance level is shown in the color legend, where red specifies the transcripts with a higher level, and blue specifies transcripts with a lower level. Bh: bromoxynil; T + Bh: tryptophan + bromoxynil; *Nm +* Bh: *Nostoc muscorum* + bromoxynil; *Ap +* Bh: *Arthrospia platensis* + bromoxynil.

The qRT-PCR analysis was used to identify and validate the genes induced in response to priming grains in different safener treatments after 24, 48, 72, 96 h, and 15 days from Bh application during the vegetation stage, either alone (Bh), or in combination with different safener treatments. Two genes related to the herbicide detoxification system were analyzed, namely *TaGS* and *TaGPX*, in addition to three genes from the *GST* gene family (*TaGSTZ, TaGSTU*, and *TaGSTL*), as shown in Figure 3, Figure 4 and Figure 5, respectively. 

The obtained results in Figure 3a showed that the *TaGS* gene was upregulated after Bh application (2.56 fold) compared with the control. By using herbicide with different safener treatments (T + Bh, *Nm +* Bh, and *Ap +* Bh), a significant increase of 3.10, 3.53, and 5.6 fold, respectively, was estimated. Also, 24 h after Bh application, the greatest increase was shown in the case of *Ap +* Bh treatment. 

Similarly, the *TaGPX* gene of the wheat plants was upregulated after Bh application, but the maximum increase of 2.31 fold was detected after 72 h (Figure 3b). The use of safener treatment with Bh resulted in a significant increase of 2.8, 3.3, and 3.69 fold for T + Bh, *Nm* + Bh, and *Ap* + Bh, respectively, and the maximum increase was again shown in the *Ap* + Bh treatment. 

Concerning the gene family of GST zeta (*TaGSTZ*), GST theta (*TaGSTU*), and GST lambda (*TaGSTL*), all of these genes showed upregulation after Bh application. The highest increase was detected after 24 h of treatment, recording 3.12, 2.8, and 1.9 fold for the three genes, respectively (Figure 4). Using T + Bh, *Nm* + Bh, and *Ap* + Bh safener treatments, a significant increase in *TaGSTZ* (5.38, 6.5, and 11.83 fold), *TaGSTU* (3.6, 4.58, and 9.2 fold), and *TaGSTL* (2.8, 3.7, and 5.3 fold) was found. According to the obtained results, the upsurge of these genes occurred 24 h after Bh application, especially in the *Ap +* Bh treatment. In conclusion, the increase in gene response upon application of Bh treatment alone could be arranged in the following sequence: GST (*TaGSTZ* > *TaGSTU* > *TaGSTL*) > *TaGS* > *TaGPX* (Figure 5).

## 3. Discussion

Crop protection against abiotic factors is essential for improvements in agricultural productivity. The use of chemical safener ingredients (such as cloquintocet mexyl, mefenpyr diethyl, fenchlorazole ethyl, tryptophan, and salicylic acid) against herbicides has been extensively reported to stimulate antioxidant metabolism, ROS signaling, and ROS detoxification processes [21] under field conditions. However, the environmental fate and possible toxicity of chemical safener compounds are poorly understood, rendering these safeners practically biohazards [22]. Therefore, delivering safeners to the crop by seed coating or targeted spraying is usually used to moderate the potential for induced environmental dangers when associating safeners with herbicides [23]. Owing to the wide variety of bioactive compounds that can be found in microalgae and cyanobacteria, the use of these microorganisms or their extracts has been shown to promote crop resistance against stress factors, such as the use of herbicides [24]. These natural remedies have long been advocated in the literature due to their potential role in improving plant growth and development in outdoor settings. Additionally, they have a low cost and are widely available. The current study investigated the effect of two cyanobacterial aqueous extracts of *A. platensis* and *N. muscorum* as natural safeners on the enzymatic and non-enzymatic antioxidant compounds in wheat plants to improve tolerance against stress conditions. 

Primed wheat grains in *A. platensis* aqueous extract increased all measured growth parameters (i.e., plant height, fresh weight, and dry weight), more than the values obtained for the *N. muscorum* aqueous extract, tryptophan, or control conditions (Table 1). In this context, our findings were consistent with many previous studies. Gheda and Ahmed [25] investigated soil inoculation with cyanobacteria, *Nostoc kihlmani,* and *Anabaena cylindrical,* enriching the soil with nitrogen, phosphorus, and potassium. Cyanobacterial inoculation stimulated germination, root and shoot lengths, dry weights, and nutrient content in the tested wheat plant. Likewise, filtrates of *A. platensis,* applied by seed soaking, foliar spraying, or as a homogenate for seed coating, were all stimulating for radish plants and showed great potential for applications in horticultural and agricultural practices [26]. Additionally, Zarezadeh et al. [27] applied cyanobacterial suspensions of *N.* *muscorum*, *Wolle avaginicola*, and *Nostoc punctiforme* as biofertilizers on *Matricaria chamomilla* L. (chamomile). The authors reported increased levels of nitrogen and growth hormones. The roots and shoots were also longer under these conditions.

In the same manner, the results of the present study showed that the application of different safener treatments induced an increase in the antioxidant enzyme activities of SOD, CAT, GPX, GST, and the non-enzymatic GSH content at all estimated times (Table 2 and Figure 1). This increase was greater with T + Bh, *Nm + Bh*, and *Ap + Bh* treatment combinations than with Bh alone. This implied more activation of the plant antioxidant defense mechanism that protected the wheat from the oxidative injury caused by the Bh treatment. These results were in agreement with Osman et al. [28], who reported that priming of bean (*Vicia faba*) seeds in an *A. platensis* suspension caused enhancement of the antioxidant enzymes, which led to the amelioration of the harmful effect of the Fluazifop Super herbicide. In addition, the *A. platensis* aqueous extract was found to contain many antioxidant compounds, including phenolics and flavonoids, to which differential antioxidant, antimicrobial, herbicide, and pesticide properties may be attributed [29]. The antioxidant defense network in the plant depends on endogenous enzymatic and non-enzymatic antioxidants. The role and efficacy of the first-line defense antioxidants, including, SOD, CAT, and GPX, are essential in the full defense approach of antioxidants [30,31]. The overexpression of different isoforms of SODs (Cu-ZnSODs, FeSODs, and MnSODs) together with scavenging H_2_O_2_ enzymes, such as CAT, will offer improved results in stress resistance [32].

Moreover, Kumar et al. [33] stated that GSH is an active redox compound that assists in keeping a homeostatic balance of the cellular redox state and is critical in plant defense mechanisms against stresses. Glutathione is a small intracellular thiol molecule that is recognized as a strong non-enzymatic antioxidant. It regulates multiple metabolic functions, avoids the oxidative denaturation of proteins under stress conditions by protecting their thiol groups, and acts as a substrate for both GPX and GST, via acting as a phytochelatin precursor for xenobiotics [34]. Furthermore, the role of GPX enzyme activity, as well as glutathione reductase (GR), accelerates tolerance against abiotic and biotic stresses in *T. aestivum* plants via the ascorbate–glutathione cycle [35,36].

Many studies suggest that GSTs protect cells against chemical-induced toxicity and provide tolerance by catalyzing S-conjugation between the thiol group of GSH and toxic metals/metalloid substrates [37]. After conjugation, the conjugate is either impounded into the vacuoles or exported from the cells by putative membrane ATP-dependent pump systems [38]. All of these functions could explain a proposed mechanism by which the investigated wheat plants could overcome the toxic effects of Bh application. Consistent with the results obtained in this study, Chronopoulou et al. [39] stated that a strong relationship was observed between the efficiency of a safener and its capability to induce GST activity. Therefore, herbicide tolerance is an outcome of the induced ability to detoxify the herbicide through GSH conjugation.

The qRT-PCR analysis was used to detect the responses of five antioxidant-related genes in response to Bh alone or in combination with tryptophan or cyanobacterial treatments (Figure 3, Figure 4 and Figure 5). According to our results, the genes *TaGS* and *TaGPX*, as well as the three genes of the GST family, *TaGSTZ, TaGSTU*, and *TaGSTL*, were upregulated during the different testing times at the vegetation stage after Bh application, especially after 24 and 72 h. Although the values of these enzymes decreased after 15 days of Bh application, they were still high for *Ap* + Bh and *Nm* + Bh, followed by T + Bh, compared with the Bh treatment alone and control plants. In this regard, the following stated functions of GSTs classes may explain their significant upregulation presented in this study. Tau and phi are the most abundant classes of GSTs in plants, playing a major role in xenobiotic metabolism [33]. The tau class has shown a potential role in protecting against oxidative damage, chemical toxicity, and physical stress agents [40]. The smaller theta and zeta classes seem to be like maleylacetoacetate isomerase (MAAI), the enzyme that is responsible for converting the cis double bond in maleylacetoacetate to the trans double bond in fumarylacetoacetate. This reaction requires GSH as a cofactor [41] By converting their serine residues to cysteine, lambda and DHAR act as thiol transferases [42]. 

Many studies are in agreement with the results of this study. Taylor et al. [9] reported that an identical subset of GSTs derived from the tau, phi, and lambda classes accumulated in wheat plant foliage when they were spray-treated with three chemical safeners, namely cloquintocet mexyl, mefenpyr diethyl, and fenchlorazole ethyl. Several authors recommended these safener compounds to enhance tolerance against aryloxyphenoxypropionate (AOPP) herbicides, which inhibit the functioning of the essential enzyme acetyl CoA carboxylase in plants. The expression of stress-inducible *GmGSTU4*, *CsGSTU1*, and *CsGSTU2* transcripts in transgenic tobacco plants enhanced tolerance against diphenyl ether herbicide, salinity, and drought stresses [43]. Most of the genes encoding detoxification enzymes, including P450s, GSTs, and UGTs, were upregulated by fluxofenim, a chemical safener in sorghum [44]. Also, Brazier-Hicks et al. [45] treated rice seedlings with metcamifen and cyprosulfamide, which were used to protect rice from the clodinafop-propargyl herbicide. The authors found that many enzymes, such as CYP, GST, and UGT, had upregulated expressions as a response [46]. 

**Figure 6:**
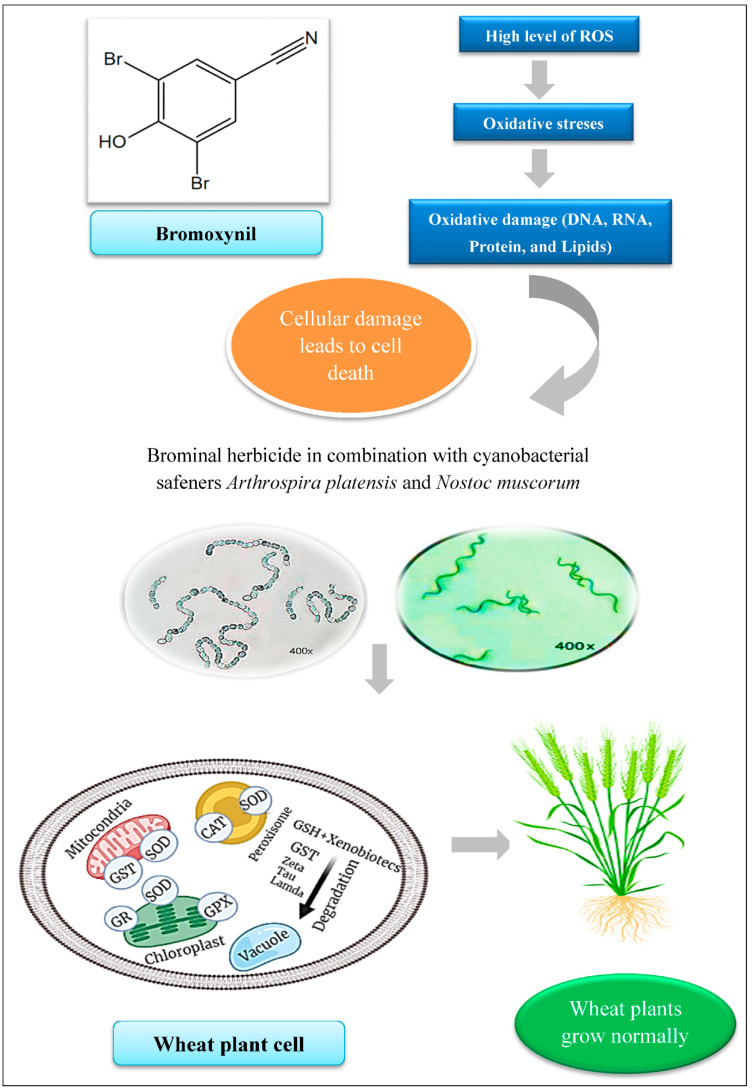
A schematic representation of the effect of cyanobacterial extract safeners against the herbicide’s harmful effect on wheat plants.

Notably, the results obtained by using qRT-PCR analysis were consistent with the results estimated by the biochemical analysis of the same enzymes, which emphasized the role of the GHS molecule as the detoxification route for Bh herbicide during the vegetation period of wheat growth. The current findings established a synergistic signal between the relative expression of wheat antioxidant-related genes and improvement in their tolerance against herbicide toxicity (Figure 6). This, in turn, triggered growth enhancement and antioxidant defense system stimulation, which improved wheat plant productivity even in the presence of Bh herbicide. 

## 4. Materials and Methods

### 4.1. Cyanobacteria Isolation, Identification, and Growth Culturing

Two cyanobacterial species were isolated from soil samples of a previously cultivated wheat plant. The two purified species were identified as *A. platensis* (Gomont) and *N. muscorum* (Calothrix C. Agardh ex Bornet and Flahault) using morphological and taxonomic features, according to Evans and Prescott [47]. The identification was also confirmed using the AlgaeBase website (http://www.algaebase.org, accessed on 20 June 2021). At the beginning of the stationary growth phase, the cultures were harvested by centrifugation at 4000 rpm (Fisher Centerific TM Centrifuge) for 10 min. Pellet cells were rinsed three times and re-suspended in sterilized distilled water to remove traces of growth medium [48]. After biomass washing, the supernatant was discarded, and 100 mL of distilled water was added to the pellet obtained from each culture and kept at 25 °C for 48 h on a shaker (VS-8480) to stimulate the extraction of secondary metabolites from cells. Thereafter, the supernatants were collected by centrifugation, as previously mentioned, and dried in a freeze dryer (LGJ-10) for 12–15 h. Then, the lyophilized powder was stored at −20 °C until use. 

### 4.2. Herbicide 

The Bromoxynil-Octanoate W (24% EC) herbicide used in this study was purchased from Novarm Company, France.

### 4.3. Plant Growth and Treatments

Wheat grains (*Triticum aestivum* L. var. Giza 171) were obtained from the Main Crops Improvement Station (MCIS), Kafr El-Sheikh City, Egypt. Uniform grains of wheat were selected and sterilized in sodium hypochlorite solution (1%) for 15 min. The grains were thoroughly washed with distilled water and then primed (soaked) for 12 h in the following treatment solutions: (a) Distilled water was used as a negative control, (b) L-Tryptophan powder (T) (EuroMedx, France) was prepared as a 0.01% solution in distilled H_2_O, and (c) cyanobacteria (*A. platensis* and *N. muscorum*) aqueous extract was used at a concentration of 1%.

The experiment was divided into five groups. Three groups were designated for the previously mentioned treatments, in addition to a negative control group (without any treatments), and a positive (Bh) treatment group. In each pot, 20 grains were sown two cm deep in the soil. The pots were kept in the greenhouse (Botany Department, Faculty of Science, Tanta University) under normal conditions of daylight and temperature (28 ± 2 °C). They were regularly irrigated with tap water every 48 h. Ammonium nitrate (31.5 kg/hectare) and superphosphate (6.3 kg/hectare) chemical fertilizers were added according to the recommended doses and times by the Ministry of Agriculture of Egypt. The Bh treatment was applied after 55 days (vegetation stage) of sowing as a post-emergence foliar herbicide according to the recommended dose (100 L/hectare), which was carried out in the early morning. The plants were grown for 120 days until the flowering stage. The following codes were given for the different treatments: Bh, T + Bh, *Nm* + Bh, and *Ap* + Bh, representing bromoxynil alone, tryptophan plus bromoxynil, *N. muscorum* aqueous extract plus bromoxynil, and *A. platensis* aqueous extract plus bromoxynil, respectively.

### 4.4. Plant Samples Collection

Plant samples were collected at the vegetation stage after 24, 48, 72, 96 h, and 15 days following Bh application. All samples were stored at −80 °C (Wisecryo freezer) for further analysis.

#### 4.4.1. Growth Parameters

Five plants were chosen from each treatment and their growth parameters (plant height, fresh weight, and dry weight) were measured. 

#### 4.4.2. Biochemical Analyses 

##### Antioxidant Activity 

The enzymatic activity of superoxide dismutase (SOD), catalase (CAT), glutathione peroxidase (GPX), glutathione-s-transferase (GST), and non-enzymatic activity of reduced glutathione (GSH) were determined after 24, 48, 72, 96 h, and 15 days (vegetation stage) from Bh application. An amount of 0.5 g of plant leaves was initially mixed with 2.5 mL of cold phosphate buffer (50 mM and pH = 7.4), 1 mM ethylenediaminetetraacetic acid (EDTA), and 1 mM dithiothreitol (DTT). After that, the mixture was centrifuged at 4000 rpm (Sigma Laborzentrifugen 1K15) for 15 min, and the supernatant was eventually recovered and stored in the freezer at −80 °C until it was used to determine antioxidant activity. The activities of SOD [49], CAT [50], GPX [51], and GST [52] were evaluated using assay kits (Biodiagnostic, Egypt (Website: www.bio-diagnostic.com accessed on 28 April 2021), according to the manufacturer’s instructions. In addition, the GSH content was estimated by the method of Ellman [53]. The intensity of the formed yellow color was measured at 420 nm. The GSH content was expressed as μmol/g FW after the preparation of a calibration curve, using GSH as a standard.

#### 4.4.3. Gene Expression Analysis Using qRT-PCR

The expression levels of the five different wheat genes, including those from the GST gene family [*Zeta* (*TaGSTZ)*, *Tau* (*TaGSTU)*, *Lambda* (*TaGSTL*)], glutathione peroxidase (*TaGPX*)*,* and glutathione synthetase (*TaGS*), were determined 24, 48, 72, 96 h, and 15 days (vegetation stage) after Bh application. Leaf tissue samples stored at −80 °C were ground in liquid nitrogen, and total RNA was extracted using Qiazol reagent (Qiagen, Biotech Company, Hilden, Germany), according to the manufacturer’s instructions.

To these, 200 μL of chloroform was added per 1 mL of qiazol to tissue homogenate. Then, the tubes containing the tissue homogenate were allowed to stand for 5 min at room temperature, followed by centrifugation at 12.000 rpm for 15 min at 4 °C. The homogenate was separated into three layers; the upper layer (aqueous layer) containing RNA was pipetted and transferred into a new microcentrifuge tube. cDNA synthesis was accomplished using the COSMO cDNA synthesis kit (Willoufort) according to the manufacturer’s instructions. For reverse transcription-polymerase chain reaction (qRT-PCR), TOPrealTMqPCR 2X PreMIX (Smart Science Co., Ltd. Klong Nueng, Klong Luang, Pathum Thani, 12120, Thailand) was used. Relative expression levels of *TaGSTZ*, *TaGSTU*, *TaGSTL*, *TaGPX, TaGS,* and *GAPDH* (as the housekeeping gene) were determined using previously described primer sequences [9,10,11,12,13,14,15,16,17,18,19,20,21,22,23,24,25,26,27,28,29,30,31,32,33,34,35,36,37,38,39,40,41,42,43,44,45,46,47,48,49,50,51,52,53,54] which were purchased from Sigma-Aldrich Inc., Missouri, USA (Table 3). qRT-PCR was performed by the CFX96 Touch thermo-cycler real-time PCR system (Bio-Rad Laboratories Inc., PTE Ltd., Singapore).

The qRT-PCR program included a 5-min cycle at 95 °C followed by 40-s cycles at 95 °C for 10 s, a 15-s cycle at 60 °C, and a 30-s cycle at 72 °C. After each run, melting curves for the amplicons were measured by raising the temperature by 0.5 °C at intervals from 50 to 99 °C while monitoring fluorescence. The expression of each gene relative to that of the control was calculated following the 2^−ΔΔCt^ method [54]. Each experiment consisted of three biological replicates.

### 4.5. Statistical Analysis

Except for qRT-PCR, where three biological replicates were used, all data was normally distributed, and all results were evaluated as means ± standard deviation (SD) of five replicates (*n* = 5). Graphpad Prism 9.0.0 (Graphpad Software, San Diego, CA, USA) was used for performing the statistical analysis of the obtained data. A one-way ANOVA was applied to compare groups at a significant difference of *p* < 0.05. The heatmap of detoxification gene expressions for the herbicide was performed using R software (4.1.1), where the heatmap function of R software was applied.

## 5. Conclusions

To the best of our knowledge, the present in vivo study is the first to provide biochemical and molecular evidence underlining the protective effects of cyanobacteria, *A. platensis,* and *N. muscorum*, as natural safeners for crop plants against practice-specific herbicides. This study not only confirmed the positive role of these natural safeners over chemical safeners but also demonstrated a deferential variation between these safeners, as *A. platensis* aqueous extract was more effective as a safener than the *N. muscorum* extracts. Priming wheat grains in the aqueous extract of these cyanobacterial safeners stimulated the growth of the plants, especially after Bh spraying. In wheat plants, the induction of antioxidant-defense enzymes, such as SOD, CAT, GPX, GST, and the non-enzymatic GSH molecules, was enhanced with a special performance for *A. platensis* treatment. 

qRT-PCR analysis showed a potent mechanism by which plants could override the way for herbicide detoxification. Based on the responses of the studied genes, including GST (*GSTZ*, *GSTU*, and *GSTL*), *TaGS*, and *TaGPX*, a detoxification pathway was suggested to be used by wheat plants to mitigate the negative effects of Bh application. The outcome of this study may improve our understanding of the toxic process and develop strategies for reducing the risks of herbicides to crop production. More prospective studies are needed to screen other beneficial natural safeners, especially from microalgae and cyanobacteria members, by virtue of their benign relationship with soil and plants. Adopting advanced molecular investigations is crucial to configuring a comprehensive mechanism of action that enables the perfect usage of these natural agents for the development of agricultural practices.

## Figures and Tables

**Table 1:** Effect of different safener treatments on growth parameters of wheat plants *.

Treatments	Plant Height (cm)	Plant FW (g)	Plant DW (g)
Control	98.07 ± 13.4 ^a^	10.47 ± 0.75 ^a^	3.09 ± 0.22 ^a^
Bh	74.94 ± 11.2 ^e^	6.89 ± 0.48 ^d^	1.78 ± 0.2 ^d^
T + Bh	80.30 ± 11.5 ^d^	7.8 ± 0.25 ^c^	1.89 ± 0.1 ^c^
*Nm +* Bh	83.20 ± 12.1 ^c^	8.56 ± 0.5 ^b^	1.98 ± 0.35 ^b^
*Ap +* Bh	89.2 ± 11.8 ^b^	9.01 ± 0.6 ^b^	2.10 ± 0.15 ^b^

* Data are presented as the mean ± SD (*n* = 5). FW: fresh weight; DW: dry weight; Bh: bromoxynil; T + Bh: tryptophan + bromoxynil; *Nm +* Bh: *Nostoc muscorum* + bromoxynil; *Ap +* Bh: *Arthrospia platensis* + bromoxynil. Different letters indicated a significant difference between treatments at *p* < 0.05.

**Table 2:** Effect of Bh application alone or in combination with different safener treatments on the activity of antioxidant enzymes *.

Treatment	Time	SOD (U/g FW)	CAT (U/g FW)	GPX (µmole/g FW)	GST (U/g FW)
Control	24 h	1.43 ± 0.02 ^a^	0.89 ± 0.04 ^a^	83.90 ± 5.6 ^a^	1.36 ± 0.2 ^a^
	48 h	1.44 ± 0.01 ^a^	0.90 ± 0.05 ^a^	84.05 ± 3.2 ^a^	1.38 ± 0.1 ^a^
	72 h	1.44 ± 0.04 ^a^	0.92 ± 0.03 ^a^	84.40 ± 4.2 ^a^	1.40 ± 0.08 ^a^
	96 h	1.46 ± 0.05 ^a^	0.93 ± 0.04 ^a^	85.03 ± 2.5 ^a^	1.41 ± 0.09 ^a^
	15 days	1.39 ± 0.3 ^a^	0.86 ± 0.10 ^a^	81.23 ± 6.5 ^a^	1.29 ± 0.1 ^a^
Bh	24 h	1.68 ± 0.1 ^b^	0.95 ± 0.01 ^b^	90.38 ± 6.5 ^b^	2.70 ± 0.1 ^b^
	48 h	1.54 ± 0.03 ^b^	1.11 ± 0.03 ^a^	136.20 ± 4.9 ^b^	1.82 ± 0.03 ^b^
	72 h	1.47 ± 0.01 ^a^	1.24 ± 0.02 ^b^	160.60 ± 7.6 ^b^	1.70 ± 0.01 ^b^
	96 h	1.48 ± 0.03 ^a^	1.14 ± 0.02 ^b^	156.10 ± 8.6 ^b^	1.65 ± 0.02 ^b^
	15 days	1.46 ± 0.2 ^b^	1.14 ± 0.20 ^b^	153.40 ± 17.3 ^b^	1.80 ± 0.15 ^b^
T + Bh	24 h	2.16 ± 0.3 ^c^	1.11 ± 0.03 ^c^	98.04 ± 2.2 ^c^	0.3 ^c^ ± 3.50
	48 h	1.90 ± 0.2 ^c^	1.26 ± 0.03 ^b^	141.80 ± 6.3 ^c^	3.23 ± 0.2 ^c^
	72 h	1.89 ± 0.06 ^b^	1.47 ± 0.02 ^c^	180.40 ± 5.3 ^c^	2.96 ± 0.15 ^c^
	96 h	1.59 ± 0.03 ^b^	1.21 ± 0.02 ^c^	00.176 ± 4.7 ^c^	2.88 ± 0.06 ^c^
	15 days	1.72 ± 0.3 ^c^	1.19 ± 0.12 ^b^	174.00 ± 12.4 ^c^	2.50 ± 0.3 ^c^
*Nm +* Bh	24 h	3.76 ± 0.2 ^d^	1.15 ± 0.02 ^c^	116.80 ± 9.5 ^d^	4.81 ± 0.5 ^d^
	48 h	2.84 ± 0.1 ^d^	1.28 ± 0.3 ^b^	150.80 ± 2.8 ^d^	4.43 ± 0.2 ^d^
	72 h	2.62 ± 0.3 ^c^	1.52 ± 0.1 ^c^	188.4 ± 10.4 ^d^	4.17 ± 0.5 ^d^
	96 h	2.08 ± 0.4 ^c^	1.23 ± 0.02 ^c^	182.80 ± 12.6 ^d^	3.80 ± 0.15 ^d^
	15 days	1.96 ± 0.2 ^d^	1.22 ± 0.15 ^b^	10.6 ^d^ ± 180.40	3.40 ± 0.4 ^d^
*Ap +* Bh	24 h	5.64 ± 0.6 ^e^	1.34 ± 0.03 ^d^	125.70 ± 8.7 ^e^	5.93 ± 0.8 ^e^
	48 h	4.23 ± 0.4 ^e^	1.46 ± 0.04 ^c^	158.90 ± 4.03 ^e^	4.94 ± 0.2 ^e^
	72 h	3.73 ± 0.2 ^d^	1.66 ± 0.2 ^d^	194.80 ± 11.2 ^e^	4.78 ± 0.25 ^d^
	96 h	3.66 ± 0.5 ^d^	1.28 ± 0.02 ^c^	185.80 ± 13.8 ^e^	4.62 ± 0.3 ^d^
	15 days	3.13 ± 0.4 ^e^	1.26 ± 0.01 ^c^	182.70 ± 15.4 ^e^	4.28 ± 0.25 ^e^

* Data are presented as the mean ± SD (*n* = 5). Bh: bromoxynil; T + Bh: tryptophan + bromoxynil; *Nm +* Bh: *Nostoc muscorum* + bromoxynil; *Ap +* Bh: *Arthrospia platensis* + bromoxynil. Different letters indicated a significant difference between treatments at *p* < 0.05.

**Table 3:** Primer sequences of the studied genes.

Gene Name	Primer Sequences (5′-3′)	
Wheat zeta GST (*TaGSTZ*)	For. 5′-CCTGGATCAGCTCTTGCTCT-3′	[9]
	Rev. 5′-AATCTGGAAGCGATTGATGG-3′	
Wheat tau GST (*TaGSTU*)	For. 5′-CAA CGA GTC CCT CAT CC-3′	[9]
	Rev. 5′-GAG GGT CTT GAG GAT GTC CA-3′	
Wheat lambda GST (*TaGSTL*)	For. 5′-GCA CTG CTT CCT CAA GAT CC-3′	[9]
	Rev. 5′-GTC ACG TAC GCA ATG TCC AC-3′	
Wheat glutathione peroxidase (*TaGPX*)	For. 5′-CTA GAC TTC AGG AAC TTG-3′	[53]
	Rev. 5′-CCT AAC TAA CTC CAA CTA C-3′	
Wheat glutathione synthetase (*TaGS*)	For. 5′-GAA CCA GCA TTG ACC TAC-3′	[53]
	Rev. 5′-TAG TGA TCC ACG AAA CAA G-3′	
*TaGAPDH* (housekeeping gene)	For. 5′-GGA GGA GTC TGA GGG AAA CC-3′	[9]
	Rev. 5′-GCT GTA TCC CCA CTC GTT GT -3′	

## Data Availability

All data generated or analyzed during this study are included in this published article.
